# Angle-insensitive narrowband optical absorption based on high-*Q* localized resonance

**DOI:** 10.1038/s41598-018-33489-6

**Published:** 2018-10-15

**Authors:** Xiya Zhu, Jichao Fu, Fei Ding, Yi Jin, Aimin Wu

**Affiliations:** 1 Center for Optical and Electromagnetic Research, State Key Laboratory for Modern Optical Instrumentation, Zhejiang University, Zijingang Campus, Hangzhou, 310058 China; 2 Centre for Nano Optics, University of Southern Denmark, Campusvej 55, DK-5230 Odense, Denmark; 3 State Key Laboratory of Functional Materials for Informatics, Shanghai Institute of Microsystem and Information Technology, CAS, Shanghai, 200050 China

## Abstract

Strong optical absorption can be achieved easily based on an array of subwavelength localized resonators. The absorption bandwidth is typically wide since subwavelength metallic resonators are limited by a low quality factor (*Q*) due to their large material loss and so do dielectric counterparts owing to their weak photon binding. Here, an angle-insensitive narrowband optical absorber is suggested, which consists of subwavelength dielectric cavities buried inside a metal. Within each cavity, a special resonant mode of high *Q* can be supported, which is absorbed slowly by the metal walls as the electric field is concentrated at the cavity center and leaks slowly into the free space due to the blocking of the top metal film covering the cavities. Such a mode is excited to trap the incident wave in the optical absorption. When low-loss silver is used, one can obtain ultra-narrowband absorption with *Q* up to 487. At lower optical frequencies, the metal film needs to be punctured so that the incident wave can couple into the cavities effectively. The suggested absorption method may find its promising prospect in thermal radiation, photonic detection, optical sensing, and so on.

## Introduction

Strong optical absorption can be achieved flexibly based on various microstructures. Many efforts have been adopted in wideband and multiband absorption^1–5^, while narrowband absorption remains unexplored largely. Narrowband optical absorption has a wide range of applications, such as narrowband thermal radiation sources^6^, narrowband photonic detectors^7,8^, and high-sensitivity sensors^9,10^. Some works have been suggested to obtain narrowband optical absorption by adopting Fabry-Perot (FP) cavities^11,12^, diffraction gratings^13–16^, and photonic crystals^17–19^. For instance, in ref.^11^, a FP-resonance-based absorber made of silver (Ag) and alumina films has an absorption bandwidth as narrow as 2 nm in the near-infrared (NIR) region. And in ref.^16^, it is found that the lattice resonance of a periodic gold (Au) nanowire array above a reflective Au film can lead to narrowband NIR absorption and strong magnetic enhancement. However, most of the aforementioned narrowband absorbers are strongly of angular dependence. That is, their absorption peaks are shifted obviously by changing the incident angle. Thus the absorption bandwidth is not narrow actually when all the incident angles are taken into account. Unlike the above absorbers requiring large absorption areas/collective effects, metamaterial absorbers based on subwavelength metal/dielectric resonant units provide another flexible method for optical absorption^20–22^, of which each unit can absorb the incident wave independently and enables pixelated optical trapping. Based on localized resonance, the absorption may be insensitive to the incident angle, e.g., angular stability from 0° to 80° is demonstrated by a metamaterial absorber made of anisotropic-medium cubes in ref.^23^. However, it is difficult to achieve narrowband optical absorption with metamaterial absorbers because of the large intrinsic material loss of subwavelength metallic resonant units and the weak photon binding nature of dielectric counterparts. For example, the NIR Au metamaterial absorber demonstrated in ref.^24^ possesses wideband absorption with *Q* < 10. Recently, there have been some works to narrow the optical absorption bandwidth of metamaterial absorbers. Based on the Fano resonance of Ag asymmetric microstructures, a narrow absorption peak with an 8 nm bandwidth is obtained at wavelength 930 nm^25^. Electromagnetically induced absorption is also used to narrow the absorption bandwidth^26,27^, and an absorption bandwidth of 8 nm has been realized at 420 THz numerically in ref.^26^. In addition, an all-Ag absorber with an absorption bandwidth less than 8 nm at wavelength 920 nm is proposed by utilizing a vertical gap plasmonic mode in the deep subwavelength scale^28^. Although these works can narrow the absorption bandwidth efficiently, the absorption becomes sensitive to the incident angle and the absorption bandwidth is still not narrow enough. In this work, a special narrowband optical absorber comprised of high-*Q* metal-dielectric hybrid cavities is proposed. The absorber possesses narrower absorption bandwidth as compared to other metamaterial ones, and is almost unaffected by the incident angle.

## Results

### NIR narrowband absorption

**Figure 1:**
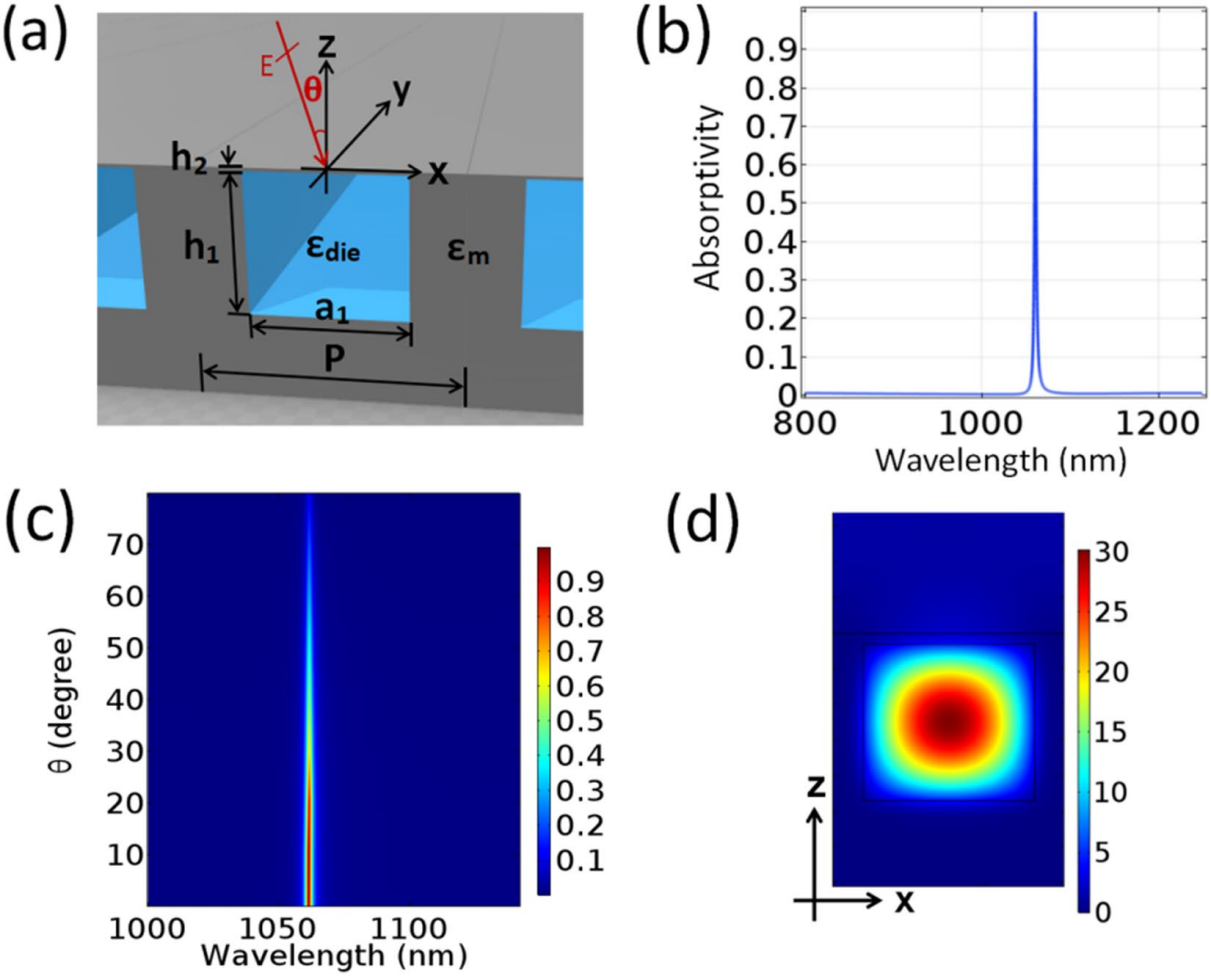
(**a**) Configuration of the designed narrowband optical absorber. (**b**) Absorption of the absorber at normal incidence, where the metal background is Ag, the rectangular cavities are filled with SiO_2_, and the geometry parameters are *a*_1_ = 480 nm, *h*_1_ = 435 nm, *h*_2_ = 30 nm, and *P* = 800 nm. (**c**) Absorption as a function of the incident angle and wavelength. (**d**) Electric field amplitude distribution in a unit at the resonant wavelength of *λ*_0_ = 1061.1 nm in (**b**), which is normalized to that of the incident electric field.

The designed optical absorber is configured in Fig. 1(a), which is composed of a periodic array of infinite-length rectangular dielectric bars buried inside a semi-infinite metal. The dielectric bars act as resonant cavities to trap the incident wave. The propagation direction of the incident wave is assumed to be fixed on the *x*-*z* plane, so the current absorption problem is simplified to a two-dimensional case, which is calculated by a commercially software.

As a demonstration, the rectangular cavities are filled with SiO_2_ of relative permittivity *ε*_die_ = 2.25, and the metal is Ag whose relative permittivity *ε*_m_ is referred to ref.^29^. The period of the cavity array is *P* = 800 nm. The dielectric cavity width and height are *a*_1_ = 480 nm and *h*_1_ = 435 nm, respectively. The left and right metal cavity walls are thick enough (*P* − *a*_1_ = 320 nm) to block the interaction of the resonant modes between the neighboring cavities, and then the absorption is weakly influenced by the width variation. The thickness of the top metal film above the cavities is chosen to be *h*_2_ = 30 nm, which is appropriate so that the incident wave can penetrate through the thin metal film and enter the cavities, and the bound fields inside the cavities are not easy to leak away into the free space. Although the light transmission is low for the current metal film thickness, incident light can still be highly trapped and absorbed if the cavity resonance is strong enough. The bottom metal substrate of the structure is thick enough to block the incident wave totally. Thus, the transmission is zero, and the absorption is calculated as *A* = 1 − *R*, where *A* is the absorptivity and *R* is the reflectivity.

The absorption of the above absorber is shown in Fig. 1(b) at normal incidence. One can see that nearly perfect absorption is achieved with absorptivity *A* = 99.99% at the resonant wavelength of *λ*_0_ = 1061.1 nm. There is only one narrow absorption peak in a wide wavelength range, and the corresponding quality factor is *Q* = 487 (defined as the ratio of the peak frequency to the full width at half maximum). The *Q* value of the current absorber is the highest compared to the other absorbers based on localized resonance whose *Q* values are commonly at the order of one hundred or less^25–28,30^. And, incident angle *θ* is varied to investigate the angular dependence of the proposed absorber, as shown in Fig. 1(c). In Fig. 1(c), one can see that the narrow absorption peak keeps fixed at the same wavelength, while the absorption decreases gradually. When incident angle *θ* becomes oblique, diffraction will appear, but it does not induce other absorption peaks as shown in Fig. 1(c). When the metal is a perfect conductor, the electric field near a metal surface for TE polarization should be zero. Although the metal is not a perfect conductor at optical frequencies, the electric field near a metal surface is still weak. The mutual coupling between the cavities is weakened by the top metal film. Due to this effect, the appearing of diffraction does not lead to additional strong absorption. As long as the cavity side walls are thick enough to prevent the coupling between the neighboring cavities, the period can be further reduced with keeping the narrowband absorption phenomenon. Additional simulation shows that the absorption peak position may be shifted by the change of the structure geometry parameters, but the angular insensitivity is maintained.

The above investigation predicts that the obtained narrowband absorption should originate from high-*Q* localized resonance. In order that one can understand the observed narrowband absorption, the electric field amplitude distribution on the *x*-*z* plane around a dielectric cavity at *λ*_0_ = 1061.1 nm for normal incidence is shown in Fig. 1(d). The electric field inside the dielectric cavity is strong, and there is no plasmon excited for the current TE polarization. Especially, the electric field is mainly concentrated at the cavity center and enhanced nearly 30 times compared to the incident wave, which indicates that the basic resonant mode of the dielectric cavity is excited strongly. The electric field is weak near the cavity sides according to the electromagnetic boundary condition as stated above, so the resonant mode is slowly absorbed by the metal walls. Thus, the resonant mode possesses a large quality factor, and the corresponding absorption peak of the absorber is very narrow. The excitation of the localized resonance is insensitive to *θ* and the mode is highly symmetric, so the absorption peak of the absorber is also angularly independent and the absorption is still strong for moderately large *θ*. This is confirmed well by Fig. 1(c).

The absorption behavior can be further understood by the temporal coupled-mode theory as an insightful way^31,32^. The designed absorber is a one-port system since there is no transmission, and according to ref.^33^, the absorption can be approximated by the following formula,

1 $${\rm{A}}=\frac{4{\gamma }_{res}{\gamma }_{rad}}{{(f-{f}_{0})}^{2}+{\gamma }_{tot}^{2}},$$

where *f*_0_ is the frequency of the basic resonant mode supported by a dielectric cavity, *γ*_rad_ is the radiative damping rate of the mode leaking into the free space, *γ*_res_ is the resistive damping rate of the mode absorbed by the lossy metal walls surrounding the cavity, and *γ*_tot_ = *γ*_res_ + *γ*_rad_ is the total damping rate. According to Eq. (1), the peak absorption is 4*γ*_res_*γ*_rad_/*γ*_tot_^2^ at *f* = *f*_0_, and perfect absorption can be achieved when *γ*_res_ = *γ*_rad_ is known as the critical coupling condition. To achieve the values of *γ*_res_ and *γ*_rad_ of the excited basic resonant mode, the two-step method described in ref.^34^ is used. Firstly, the metal is assumed to be lossless, i.e. the imaginary part of *ε*_m_ is zero. γ_rad_ can be obtained since now it is equal to the mode’s total damping rate *γ*_tot_. Secondly, the loss is added back to *ε*_m_, and a new value of *γ*_tot_ is obtained. Then, the resistive damping rate can be obtained according to *γ*_res_ = *γ*_tot_ – *γ*_rad_.

**Figure 2:**
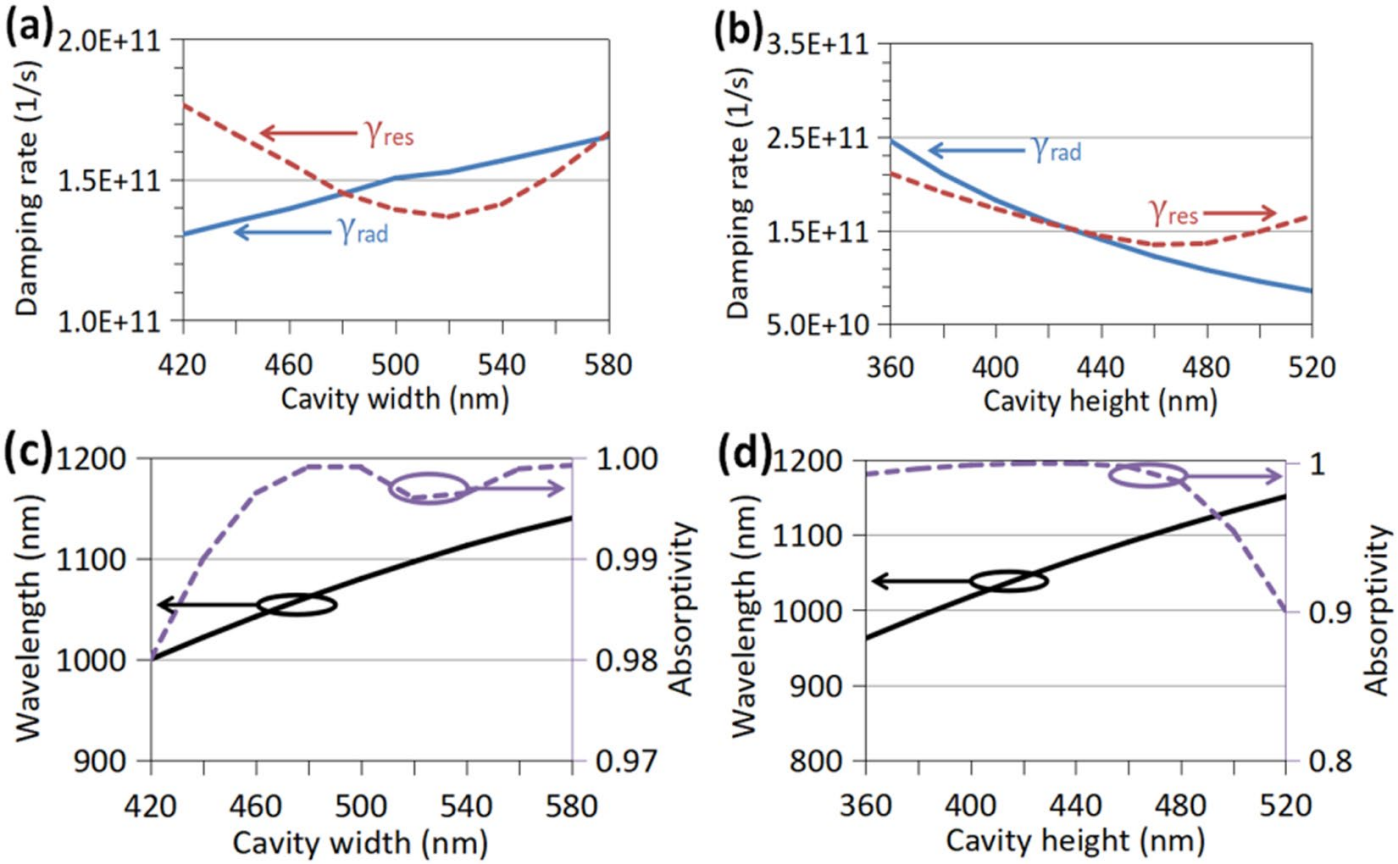
(**a**) and (**b**) Dependence of radiative damping rate *γ*_rad_ (blue solid curve) and resistive damping rate *γ*_res_ (red dashed curve) on cavity width *a*_1_ and cavity height *h*_1_, respectively. (**c**) and (**d**) Dependence of resonant wavelength *λ*_0_ (black solid curve) and absorption *A* (purple dashed curve) on *a*_1_ and *h*_1_, respectively, which is got by full-wave simulation at normal incidence.

The values of *γ*_rad_ and *γ*_res_ can be adjusted by varying the absorber’s geometry parameters to realize nearly perfect absorption. As *a*_1_ is varied with *h*_1_ = 435 nm, *h*_2_ = 30 nm, and *P* = 800 nm, the variation of *γ*_rad_ and *γ*_res_ is shown in Fig. 2(a), and that of *λ*_0_ and *A* of the optical absorber is shown in Fig. 2(c) at normal incidence which is got by full-wave simulation. As is shown in Fig. 2(a), *γ*_res_ is decreased first and then increased as the cavity width is enlarged, while *γ*_rad_ keeps increasing. When a cavity is widened, the left and right metal walls leave far away from the electric field concentrated at the cavity center so that the cavity mode is absorbed by the lossy metal walls at a lower rate. When the cavity width is increased further, as shown by Fig. S1 in the supplementary material, the cavity mode is deformed in some degree and the top and bottom metal walls may touch stronger electric field, so *γ*_res_ is increased again. Thus, the curve of *γ*_rad_ has two cross points (*a*_1_ ≈ 480 nm, and *a*_1_ ≈ 580 nm) with the U-shape curve of *γ*_res_, which both mean that the critical coupling condition can be fulfilled according to Eq. (1). This is verified by Fig. 2(c) where one can see that the absorption is nearly perfect at the two values of *a*_1_. When the incident angle becomes oblique, the absorber may leave the critical coupling condition and the absorption is reduced correspondingly. In addition, as *h*_1_ is varied with *a*_1_ = 480 nm, *h*_2_ = 30 nm, and *P* = 800 nm, the variation of *γ*_rad_ and *γ*_res_ is shown in Fig. 2(b), and that of *λ*_0_ and *A* is shown in Fig. 2(d). As shown in Fig. 2(b), *γ*_rad_ keeps decreasing and *γ*_res_ turns to increase after *h*_1_ is larger than 460 nm. The two curves of *γ*_rad_ and *γ*_res_ cross only at *h*_1_ ≈ 435 nm where the critical coupling condition is fulfilled. In Fig. 2(c,d), one can also notice that the absorption of the absorber is decreased quickly when *γ*_rad_ is departed far away from *γ*_res_. And, it is well known that when a cavity is enlarged, the resonant wavelength of the basic resonant mode will be red shifted, which is demonstrated in Fig. 2(c,d). Compared to the effect of widening the cavities, the heightening of the cavities leads to quicker red shift of *λ*_0_.

In the above investigation, low-loss Ag is used for narrowband absorption. If other high-loss metals are used instead, narrow absorption bandwidth can still be obtained, which is several times narrower than that by other localized-resonance-based absorbers made of the same metal. For example, when Au is used with *ε*_m_ referred to ref.^35^, *Q* as high as 56 can be obtained with absorption around 99.4% at *λ*_0_ = 1088 nm for normal incidence, where the parameters are optimized as *a*_1_ = 460 nm, *h*_1_ = 460 nm, *h*_2_ = 13 nm, and *P* = 650 nm. The Au film above the cavities can be thicker to reduce the practical fabrication difficulty. However, as will be discussed below, slots of appropriate width are necessary to be introduced into the metal film to help couple the incident wave into the cavities effectively.

### Middle infrared (MIR) narrowband absorption

**Figure 3:**
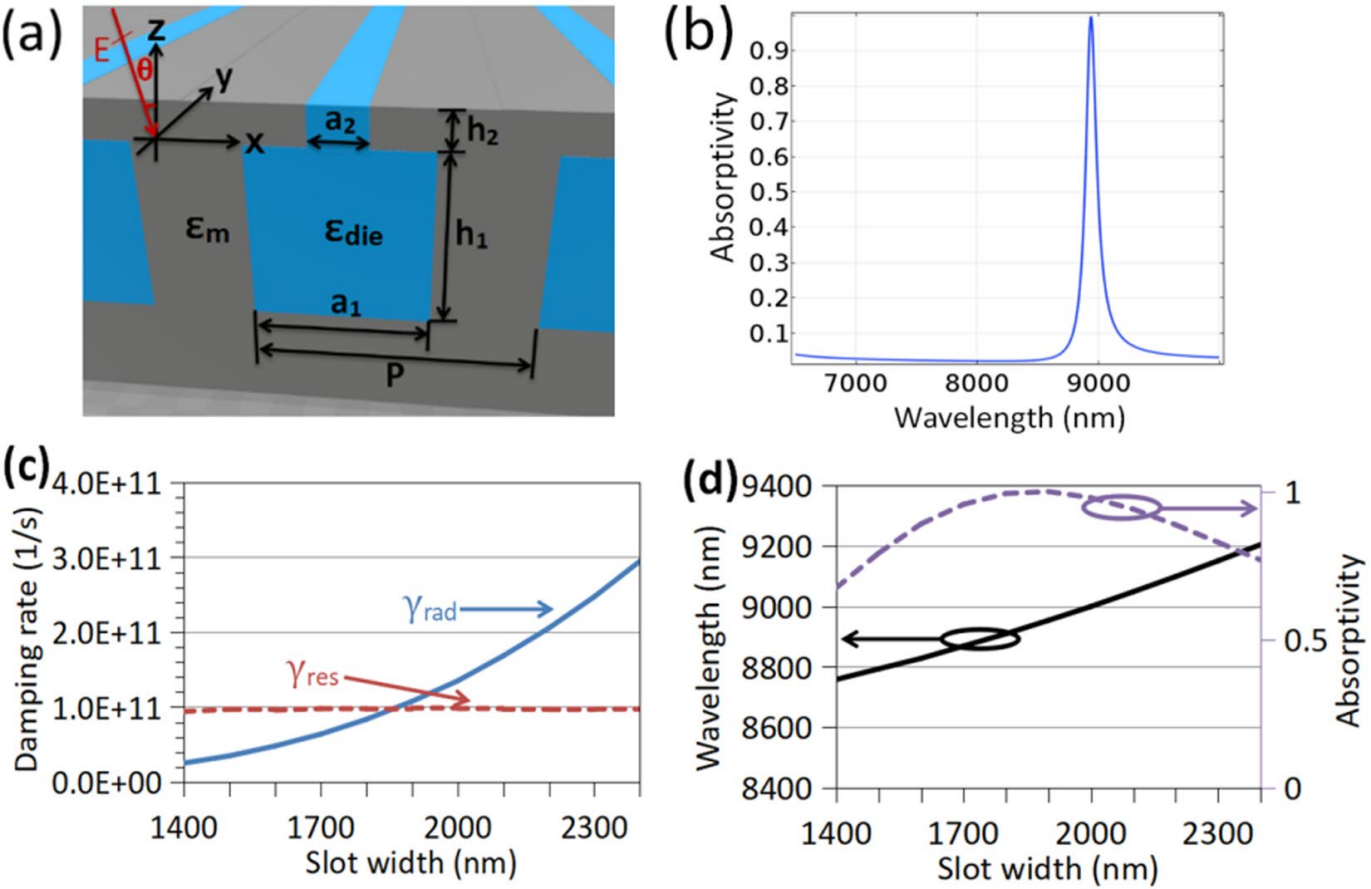
(**a**) Absorption structure with a punctured top metal film. (**b**) Absorption at normal incidence when in (**a**), the metal background is Au, the rectangular cavities are filled with SiO_2_, and the geometry parameters are *a*_1_ = 4 μm, *a*_2_ = 1.85 μm, *h*_1_ = 4 μm, *h*_2_ = 0.2 μm, and *P* = 6 μm. (**c**) Dependence of radiative damping rate *γ*_rad_ (blue solid curve) and resistive damping rate *γ*_res_ (red dashed curve) on slot width *a*_2_. (**d**) Dependence of resonant wavelength *λ*_0_ (black solid curve) and absorption *A* (purple dashed curve) on *a*_2_, which is got by full-wave simulation at normal incidence.

The suggested narrowband absorption based on high-*Q* localized resonance can also work at lower optical frequencies, e.g., MIR. However, it should be noticed that a metal acts more like a perfect electric conductor in the MIR. Therefore, the absorber in Fig. 1(a) cannot be directly scaled up to work as a MIR absorber because the top metal film becomes a good reflector to prevent strong absorption. To overcome this problem, slots with width *a*_2_ are introduced into the top metal film parallel to the axes of the below dielectric bars as shown in Fig. 3(a), which are used to enhance the coupling of the basic cavity mode inside each cavity with the incident wave. For demonstration, the metal is Au, and the dielectric filling the cavities is assumed to be of *ε*_die_ = 2.25 which can be flexibly replaced with some practical lossless MIR dielectric material. Fig. 3(b) shows the absorption of the current absorber at normal incidence with *a*_1_ = 4 μm, *a*_2_ = 1.85 μm, *h*_1_ = 4 μm, *h*_2_ = 0.2 μm, and *P* = 6 μm. The peak absorption is larger than 99.9% at *λ*_0_ = 8.94 μm, and the narrow absorption peak has a high quality factor of *Q* = 81. This *Q* value is larger than that in the NIR because now Au is more like a perfect electric conductor with lower material loss. The electric field amplitude distribution at the resonant wavelength is shown by Fig. S2 in the supplementary material, which is similar to the field distribution shown in Fig. 1(c). When the other geometry parameters are fixed, the influence of *a*_2_ on *γ*_rad_ and *γ*_res_ is shown in Fig. 3(c), and that on *λ*_0_ and *A* is shown in Fig. 3(d). One can see that the radiative damping rate goes up exponentially since the bound mode in each cavity is easier to leak as the slot is widened, and it is increased 10 times larger as the slot width is increased from 1.4 μm to 2.4 μm, but the resistive damping rate is nearly unaffected. The critical coupling condition is fulfilled at *a*_2_ ≈ 1.85 μm where the absorption is nearly perfect as shown in Fig. 3(d). And, according to our simulation which is not shown here, the MIR absorber also possesses good angular insensitivity.

## Discussion

Dielectric cavities embedded inside a metal have been found to support a high-*Q* resonant mode. Such localized resonance has been used to effectively narrow the absorption of the suggested angle-insensitive optical absorber. The absorption bandwidth is several times narrower than that of common localized-resonance-based absorbers, no matter low-loss or high-loss metals are used. The suggested absorber can operate at various optical frequencies, and the top metal film is necessary to be punctured when it operates at lower optical frequencies. Some special micro-manufacturing technologies are needed to fabricate the absorption structure. To simplify the fabrication, a compromised absorber may be constructed by putting a periodic array of dielectric bars on a metal substrate, each of which is capped with a metal coating. Angle-insensitive narrowband optical absorption can be still realized although the bandwidth is widened in some degree.

## Methods

Comsol Multiphysics is used for the simulation. A single unit cell of the cavity array is simulated, and a periodic condition is set at the left and right boundaries. In all the simulation, TE-polarized wave (the incident electric field is polarized along the *y*-axis) is incident from the top.

## Electronic supplementary material

Supporting Information
