# Supplementary material for: Angle-insensitive narrowband optical absorption based on high-Q localized resonance
Source: Sci Rep. 2018 Oct 15;8:15240. doi: 10.1038/s41598-018-33489-6 (PMC6189117; doi:10.1038/s41598-018-33489-6)
Supplement: Supplementary file 1 — Supporting Information [file 41598_2018_33489_MOESM1_ESM.docx]

Supporting Information for:

**“Angle-insensitive narrowband optical absorption based on high-*Q* localized resonance”**

Xiya Zhu,^1^ Jichao Fu,^1^ Fei Ding,^2^ Yi Jin,^1,3,*^ and Aimin Wu^3,*^

1. **Influence of cavity geometry parameters on the localized field distribution in resonance-enhanced absorption**


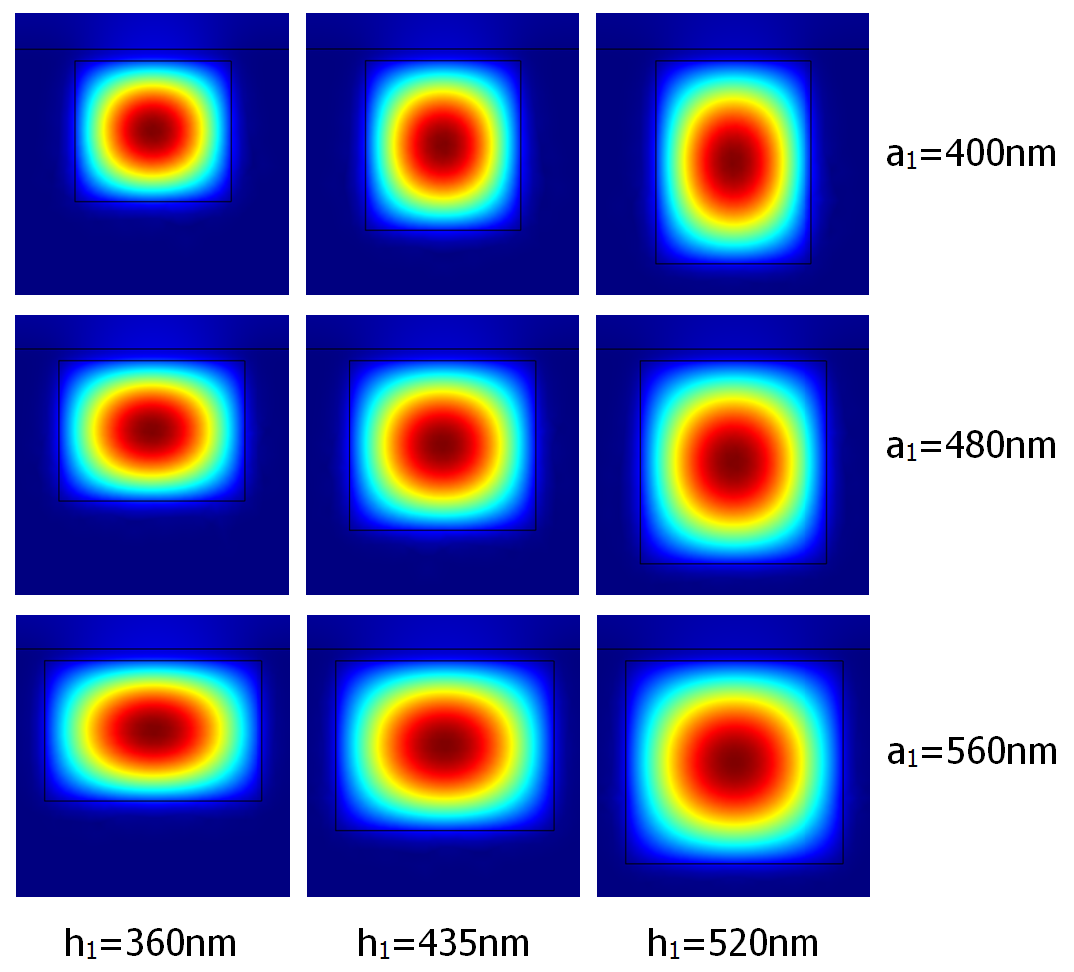


Fig. S1. Electric field amplitude distribution at the resonant wavelength for different cavity geometry parameters. The other absorber parameters are given in Fig. 2.

1. **Localized field distribution of the MIR narrowband absorber**


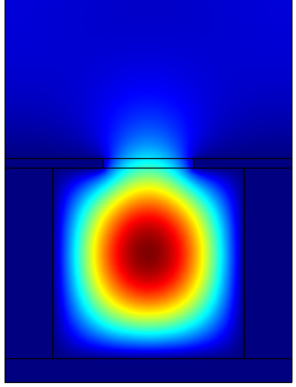


Fig. S2. Electric field amplitude distribution corresponding to the resonant peak in Fig. 3(b).
